# Significantly Longer Shedding of Norovirus Compared to Rotavirus and Adenovirus in Children with Acute Gastroenteritis

**DOI:** 10.3390/v15071541

**Published:** 2023-07-13

**Authors:** Yuanyuan Qiu, Stephen B. Freedman, Sarah Williamson-Urquhart, Ken J. Farion, Serge Gouin, Naveen Poonai, Suzanne Schuh, Yaron Finkelstein, Jianling Xie, Bonita E. Lee, Linda Chui, Xiaoli Pang, on behalf of the Pediatric Emergency Research Canada Probiotic Regimen for Outpatient Gastroenteritis Utility of Treatment (PROGUT) Trial Group

**Affiliations:** 1 Department of Laboratory Medicine and Pathology, University of Alberta, Edmonton, AB T6G 2R3, Canada; 2 Sections of Pediatric Emergency Medicine and Gastroenterology, Departments of Pediatrics and Emergency Medicine, Cumming School of Medicine, University of Calgary, Calgary, AB T3B 6A8, Canada; 3 Paediatric Emergency Research Team, Alberta Children’s Hospital, University of Calgary, Calgary, AB T3B 6A8, Canada; 4 Departments of Pediatrics and Emergency Medicine, University of Ottawa, Ottawa, ON K1H 8L1, Canada; 5 Division of Paediatric Emergency Medicine, Department of Pediatrics, Centre Hospitalier Universitaire Ste-Justine, Université de Montréal, Montréal, QC H3T 1C5, Canada; 6 Division of Pediatric Emergency Medicine, Departments of Pediatrics, Internal Medicine, Epidemiology & Biostatistics, Schulich School of Medicine and Dentistry, London, ON N6A 5W9, Canada; 7 Division of Paediatric Emergency Medicine, The Hospital for Sick Children, University of Toronto, Toronto, ON M5G 1X8, Canada; 8 Divisions of Emergency Medicine and Clinical Pharmacology and Toxicology, Research Institute, Hospital for Sick Children, University of Toronto, Toronto, ON M5G 1X8, Canada; 9 Department of Pediatrics, Cumming School of Medicine, University of Calgary, Calgary, AB T3B 6A8, Canada; 10 Department of Pediatrics, Faculty of Medicine & Dentistry, Women and Children’s Health Research Institute, Stollery Children’s Hospital, University of Alberta, Edmonton, AB T6G 2R3, Canada; 11 Alberta Precision Laboratory, Public Health Laboratory, Edmonton, AB T6G 2J2, Canada

**Keywords:** acute gastroenteritis, norovirus, rotavirus, adenovirus, viral load, fecal shedding, children

## Abstract

Worldwide, acute gastroenteritis (AGE) is a major cause of morbidity and mortality in children under 5 years of age. Viruses, including norovirus, rotavirus, and enteric adenovirus, are the leading causes of pediatric AGE. In this prospective cohort study, we investigated the viral load and duration of shedding of norovirus, rotavirus, and adenovirus in stool samples collected from 173 children (median age: 15 months) with AGE who presented to emergency departments (EDs) across Canada on Day 0 (day of enrollment), and 5 and 28 days after enrollment. Quantitative RT-qPCR was performed to assess the viral load. On Day 0, norovirus viral load was significantly lower compared to that of rotavirus and adenovirus (*p* < 0.001). However, on Days 5 and 28, the viral load of norovirus was higher than that of adenovirus and rotavirus (*p* < 0.05). On Day 28, norovirus was detected in 70% (35/50) of children who submitted stool specimens, while rotavirus and adenovirus were detected in 52.4% (11/24) and 13.6% (3/22) of children (*p* < 0.001), respectively. Overall, in stool samples of children with AGE who presented to EDs, rotavirus and adenovirus had higher viral loads at presentation compared to norovirus; however, norovirus was shed in stool for the longest duration.

## 1. Introduction

Worldwide, acute gastroenteritis (AGE) is one of the leading causes of morbidity and mortality of children younger than 5 years [1,2]. AGE cases are predominantly caused by viruses, most commonly norovirus, rotavirus, and enteric adenovirus [3]. Despite the fact that the majority of virus-related AGE infections are self-limiting, they still significantly contribute to the burden of disease in many countries [1].

Human norovirus is the leading etiology of non-bacterial AGE among individuals of all ages globally [1,2,3], and is responsible for 60% of AGE cases in the United States, accounting for 400,000 emergency department (ED) visits and 71,000 hospitalizations each year [4]. Norovirus is highly contagious due to its low infective dose, various transmission routes, and persistence in the environment. Noroviruses were recently grouped into ten genogroups (GI-GX), with GII being the most predominant [5]. Infected individuals can shed over 10^9^ copies of viral particles in one gram of feces [6]. In immunocompetent individuals, norovirus infection is usually self-limiting, with clinical symptoms lasting for 2–3 days [6,7].

Rotavirus was the most common cause of AGE in infants and young children worldwide before the introduction of the rotavirus vaccine. According to the Global Burden of Disease report, rotavirus infection caused 128,500 deaths and more than 250 million episodes of diarrhea among children younger than 5 years in 2016 [8]. Although the rotavirus vaccine has substantially reduced its incidence in countries with high vaccine coverage [9,10], rotavirus remains a major cause of AGE in children. Although most children with rotavirus gastroenteritis have high amounts of virus in their feces, shedding usually ceases within 10 days of symptom onset [10].

Adenovirus consists of non-enveloped, double-stranded DNA viruses, and belongs to the family of *Adenoviridae*. In humans, there are 88 serotypes in seven species (species A–G) [11]. Enteric adenovirus, which classically refers to serotypes 40 and 41 of specie F, was estimated to be responsible for over 52,000 deaths globally among children younger than 5 years in 2016, behind only rotavirus and Shigella [1]. The incubation period for adenovirus, typically 8–10 days, is longer than that of norovirus and rotavirus, and the illness can last as long as two weeks [12,13].

Although it is known that viruses can be shed for an extended period after the resolution of acute symptoms, the duration of virus shedding in stool varied among different studies [14,15]. Few studies have reported the kinetics and duration of viral shedding in stool samples of children with AGE infected with norovirus, rotavirus, and adenovirus [16]. In this prospective cohort study, we evaluated the viral load and virus shedding in stool samples collected from children with AGE at an index ED visit and during follow-up 5 and 28 days after the visit using real-time reverse transcription quantitative PCR (RT-qPCR).

## 2. Materials and Methods

### 2.1. Study Design

This study was part of the Probiotic Regimen for Outpatient Gastroenteritis Utility of Treatment (PROGUT) study, which found no overall or virus-specific clinical benefits associated with probiotic therapy in children who presented to the ED with AGE as compared to a placebo [17,18]. In addition, probiotic therapy was not associated with any reduction in viral shedding in stool [18]. We performed a secondary analysis focused on stool virus shedding to assess and compare the kinetics and duration of norovirus, rotavirus, and adenovirus shedding in children with AGE.

Five Canadian tertiary pediatric centers participated in this study. Children aged 3 to 48 months who presented to the ED with ≥3 diarrheal stools in a 24 h period and had a duration of illness of less than 72 h were eligible to participate. Attempts were made to collect stool specimens from all participants on Day 0 (ED enrollment), and 5 and 28 days later. Only participants who provided stool specimens at multiple time points in this study and tested positive for norovirus, adenovirus, or rotavirus were included in this analysis.

### 2.2. Study Objective and Outcome Measures

We sought to evaluate the viral load and duration of norovirus, rotavirus, and adenovirus shedding in children who presented to the ED with acute diarrhea. The study outcome was to compare the viral load and detection rate of norovirus with rotavirus and adenovirus on Days 0, 5, and 28. The Modified Vesikari Scale (MVS), with a maximum 20-point score, was used to quantify disease severity over a broad range of symptoms and interventions [19,20]. This scale has been employed in several studies, and effectively measured overall disease severity in children with AGE [17,18,21]. In this study, MVS scores were only available for Day 0.

### 2.3. Specimen Collection

Participants were enrolled into the trial between 5 November 2013, and 7 April 2017. Study participants were asked to provide a stool sample on Day 0 prior to ED discharge. If a specimen was not provided prior to ED discharge, caregivers were instructed to collect a stool sample at home, which was retrieved by a study-funded courier service.

Day 5 and Day 28 stool samples were requested from all study participants who provided a Day 0 stool sample, either while in the ED or at home. Caregivers were provided with collection instructions along with containers. Specimens were labeled with the date and time of collection and the subject’s study identification number. They were returned to the research team by a study-funded courier service within 12 h of collection. All specimens were placed in coolers with ice packs while in transit to the laboratory. Upon receipt, each sample was split and frozen for future testing. Sites then batch-shipped all frozen stool samples to the Alberta Public Laboratories-ProvLab (Edmonton, AB, Canada) bi-annually to enable interim laboratory analyses to verify collection and processing procedures.

### 2.4. Detection of Gastroenteritis Viruses

Total nucleic acid was extracted from 100–150 mg solid or 100 µL liquid stool using NucliSENS^®^ easyMag^®^. Nucleic acid extracts were tested using the Luminex xTAG^®^ Gastrointestinal Pathogen Panel (GPP) (Toronto, ON, Canada) that detects norovirus GI and GII, rotavirus, adenovirus 40/41, and 11 non-viral pathogens, as per the manufacturer’s instructions. Day 5 and 28 specimens were tested only if the Day 0 specimen tested positive for a pathogen.

### 2.5. Virus Quantification

The serial Day 0, 5, and 28 stool samples with norovirus, rotavirus, and/or adenovirus detected using the Luminex xTAG GPP assay were further tested using RT-qPCR to quantify the viral load in the sample. A 10% (weight/volume) stool solution was prepared with phosphate-buffered saline (PBS) and centrifuged at 12,000 rpm for 5 min at 4 °C. Two hundred µL of the supernatant was used for nucleic acid extraction using a MagaZorb RNA extraction kit (Promega, Madison, WI, USA) according to the manufacturer’s instructions; the volume of the final elution was 50 µL. Two-step reactions, including reverse transcription and qPCR, were performed to detect norovirus, rotavirus, and adenovirus using an ABI 7500 fast sequence detection system (Applied Biosystems, Foster City, CA, USA), as previously described [22,23,24]. An individual qPCR reaction was performed for each virus using probes all labeled with FAM and TAMRA dyes. Ten-fold serial dilutions from 100 copies to 1 × 10^6^ copies of DNA fragments of each virus were used to establish the external standard curve for viral quantification [23,24]. Virus load was expressed as genome equivalent (GE) copies/gram of stool.

### 2.6. Statistical Analysis

Viral load quantification values were log10 transformed to make data conform to normality. To compare viral load among viruses collected on Day 5, we conducted a multivariable linear regression model using viral load on Day 5 as the dependent variable. Covariates in the model included the type of virus infected (with norovirus being the reference group), age, baseline duration of symptoms, baseline MVS score, and Day 0 viral load. To compare viral load between the viruses collected on Day 28, we conducted the same regression model, with norovirus viral load on Day 28 being the dependent variable. All analyses were specified a priori and were two-sided; statistical significance was set at *p* < 0.05. We included data from all participants who met study eligibility criteria and provided the required specimens. Analyses were performed using SPSS 26.0 (Armonk, NY, USA: IBM Corp).

The Pearson correlation coefficient (r) was calculated to measure the correlation between viral load and disease severity (MVS score) on Day 0.

## 3. Results

### 3.1. Norovirus Shedding in Stool Samples

**Figure 1:**
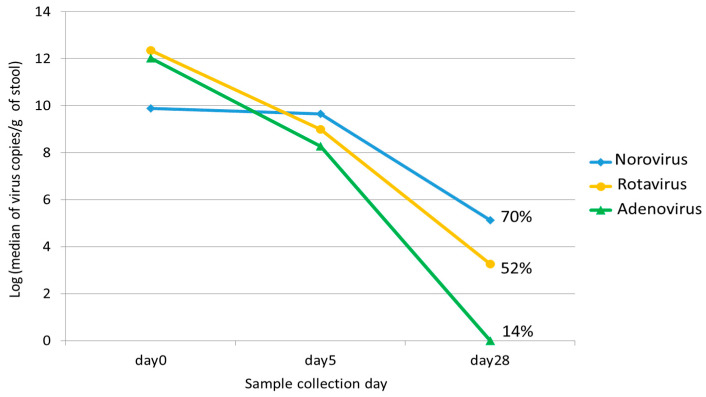
The median viral loads of norovirus, rotavirus, and adenovirus on different collection days (Days 0, 5, and 28). The percentage on Day 28 represents the positive detection rate for each virus in stool.

Data for children who tested positive for norovirus, rotavirus, or adenovirus and provided serial stool samples are summarized in Table 1. Seventy-nine children tested positive for norovirus, with a median age of 13 months (IQR: 9.5, 20.5). Norovirus GII was detected in 78 patients, and norovirus GI in 1 patient. The median viral load of norovirus was 9.9 log GE copies/g stool on Day 0 and 9.7 log GE copies/g stool on Day 5. On Day 28, norovirus was detected in 70.0% (35/50) of specimens, with a median viral load of 5.1 log GE copies/g stool (Table 2, Figure 1 and Figure S1A).

### 3.2. Rotavirus Shedding in Stool Samples

The median age of the 52 children who tested positive for rotavirus was 20 months (IQR: 12.8, 31.8). The median viral load of rotavirus was 12.3 log GE copies/g stool on Day 0 and 8.8 log GE copies/g stool on Day 5. On Day 28, rotavirus was detected in 52.4% (11/21) of specimens with a median viral load of 3.3 log GE copies/g stool (Table 2 and Figure 1 and Figure S1B).

### 3.3. Adenovirus Shedding in Stool Samples

Forty-two children with a median age of 13 months (IQR: 9, 20.5) tested positive for enteric adenovirus 40/41. The median viral load of adenovirus was 12.1 and 8.4 log GE copies/g stool on Days 0 and 5, respectively. Adenovirus was detected in 13.6% (3/22) of specimens on Day 28 (Table 2 and Figure 1 and Figure S1C).

### 3.4. Comparison of Viral Load and Detection Rate among the Three Viruses

Multivariable linear regression showed a lower viral load of norovirus on Day 0 compared to rotavirus and adenovirus (*p* < 0.001) (Table 3). However, the median viral loads of norovirus were higher than rotavirus and adenovirus on Days 5 and 28 (*p* < 0.001) (Table 3). In terms of detection rate on Day 28, norovirus was detected in more children compared to rotavirus and adenovirus (*p* < 0.001). Adenovirus had the lowest detection rate on Day 28 among the three viruses (*p* < 0.05). The Pearson correlation coefficient showed no association between the Day 0 viral load and the MVS score for norovirus (r = 0.18, *p* = 0.13), a low correlation for rotavirus (r = 0.29, *p* = 0.05), and a moderate correlation for adenovirus (r = 0.4, *p* = 0.01).

### 3.5. Co-Infection of Mixed Viruses in Children with AGE

Stool samples collected from 10 children had more than one virus detected, including four with adenovirus/rotavirus, four with norovirus/rotavirus, and two with adenovirus/norovirus (Table 4). Nine of the ten (90%) specimens collected from children with mixed viral infection had a dominant viral pathogen which had a higher viral load on Day 0 and Day 5.

## 4. Discussion

In this prospective study of children who presented to the ED with acute infectious diarrhea, we examined the kinetics of viral shedding in stool samples of three major gastroenteritis viruses—norovirus, adenovirus, and rotavirus. All three viruses were shed in high levels at the time of presentation for ED care when the first specimen was obtained, and demonstrated unique fecal shedding kinetics. Norovirus had the lowest viral load on Day 0 among the three viruses, but it shed longer and at a higher viral load compared to rotavirus and adenovirus on Days 5 and 28.

Among the children recruited with acute diarrhea who tested positive for one of the three viruses, norovirus was detected in 46%, followed by rotavirus (30%) and adenovirus (24%). The peak shedding of norovirus has been reported to occur between symptom onset to 5 days after infection [25], with over 10^9^ copies of viral particles shed in one gram of feces [6]. Cheng et al. reported that among hospitalized pediatric patients, norovirus viral load increased between days 2 and 9 following illness onset, and most norovirus shedding in feces ceased by day 15, with an average viral load of 7.25 log genome copies/mL for all samples [26]. In our study, we did not observe an increase in viral load after illness onset, but the median viral load of norovirus on Day 0 was 9.9 log GE copies/g stool, and it remained persistently high on Day 5 (9.7 log GE copies/g stool). On Day 28, 70% of children still tested positive for norovirus, with a median viral load of 5.1 log GE copies/g stool. This is consistent with previous reports that younger children shed norovirus in stool for a longer period, especially infants less than 6 months of age, who have been reported to shed detectable virus for a median of 42 days compared to just 10 days among patients older than 1 year of age [27]. Human challenge studies also report that norovirus shedding can continue for up to 60 days after symptoms disappear [6,28,29]. Norovirus reinfection is often defined as a positive result 14 days after a previous positive test. Our results support the notion that use of a 14-day interval to define reinfection is inadequate given the longer duration of shedding in young infants. Further studies of norovirus shedding with a larger sample size and among different age groups are needed to characterize the optimal time frame to define incidence of norovirus infection.

In recent years, RT-qPCR has become the gold standard for rapid, sensitive, and specific detection of norovirus in clinical samples [30]. However, a limitation of molecular assays is the inability to differentiate between infectious and non-infectious virus (i.e., to determine transmissibility); thus the communicable period of norovirus is unclear, and this has major implications for infection prevention and control practices. Chan et al. reported that a RT-qPCR cycle threshold (Ct) cut-off of 30 might define the lower limit of transmissibility, based on experiments conducted with human intestinal enteroid cultures inoculated with GII.4 Sydney[P31]-infected fecal samples [31]. However, due to the lower sensitivity of viral culture systems for norovirus, the variable replication efficiencies of different norovirus genotypes in the enteroid system, and inter-laboratory variation in RT-qPCR assays, it is possible that norovirus in stool samples with a Ct value >30 might still be transmissible.

The median rotavirus viral load of 12.3 log GE copies/g stool detected on Day 0 in our study aligns with a previous report [32]. We also found that compared to norovirus, rotavirus had a higher viral load on Day 0, but lower viral loads on Days 5 and 28. Moreover, we found that 52% of children tested positive for rotavirus on Day 28, which was lower than the 70% detection rate of norovirus on Day 28. However, prolonged rotavirus shedding of up to 57 days after symptom resolution has been reported [33,34]. Although the ability to culture rotavirus was established in the 1980s [35], no studies examining the infectivity of rotavirus in stools had been undertaken to define the period of transmissibility.

Few studies have reported the kinetics of viral load and shedding of adenovirus in stool samples [36,37], especially for comparison with other gastroenteritis viruses. The available literature reports that adenovirus 40/41 had higher viral loads compared to non-enteric adenovirus in children with AGE [36]. We report a high viral load of adenovirus on Day 0, with a rapid decline in virus level on Days 5 and 28. Compared to norovirus and rotavirus, adenovirus had the lowest detection rate on Day 28 (13.6%), indicating that the duration of adenovirus 40/41 shedding in stool is shorter than norovirus and rotavirus.

Although previous studies reported that a higher stool viral load was associated with severe clinical symptoms for norovirus infection in cancer patients at the time of diagnosis [38], we did not identify a significant correlation between norovirus viral load and disease severity on Day 0. A moderate correlation between adenovirus viral load and MVS score was observed in this study, which was in agreement with a previous finding that children infected with adenovirus with higher viral loads had more severe disease [37]. For rotavirus, it was reported that a higher level of fecal viral load was positively associated with disease severity in children [34]. We also identified a low correlation between the Day 0 rotavirus viral load and MVS score. As MVS scores were not available for Days 5 and 28 in this study, it was not clear whether there was an association between viral load and disease severity on those days.

Some limitations merit mention. We did not sample specimens between Day 5 and Day 28; therefore, the kinetic of viral load and shedding during this time period was not clear. In addition, this study enrolled a cohort of otherwise healthy young children [18]; thus, the findings could not be extrapolated to the kinetics of virus shedding among immunocompromised children and those > 48 months of age.

## 5. Conclusions

In conclusion, rotavirus and adenovirus had higher viral loads at the illness onset compared to norovirus; however, norovirus demonstrated the longest duration of virus shedding, with moderately high viral loads in the stools of young children up to Day 28 after presenting to EDs due to acute infectious diarrhea. The long duration of detection of the gastroenteritis virus in stool samples highlights the importance of further studies regarding the infectivity of qPCR positive samples and the need to revisit the definition of incident infection.

## Figures and Tables

**Table 1:** Number of serial stool samples collected on different days that were norovirus-, rotavirus-, and adenovirus-positive.

	Adenovirus	Norovirus	Rotavirus
Day 0, Day 5, and Day 28 serial stool samples, n	66	129	54
Day 0 and Day 5 serial stool samples, n	40	58	62
Day 5 and Day 28 serial stool samples, n	0	14	6
Total stool samples, n	106	201	122
Total children, n	42	79	52

**Table 2:** Norovirus, rotavirus, and adenovirus viral loads in different groups of serial stool samples based on availability of samples for qPCR tests.

	Viral Load (Log GE Copies/g Stool)	Serial Samples			Serial Samples		Serial Samples	
		Day 0	Day 5	Day 28	Day 0	Day 5	Day 5	Day 28
Adenovirus	Median (range)	11.7 (5.3–13.3)	8.1 (4.4–11.8)	0 (0–8.2)	12.3 (4.3–13.3)	9.0 (0–12.0)	N/A	
Norovirus	Median (range)	9.9 (0–10.8)	9.6 (0–10.6)	5.1 (0–7.6)	9.9 (5.5–10.8)	9.5 (0–10.3)	10.3 (9.6–11.2)	5.2 (0–7.3)
Rotavirus	Median (range)	12.3 (0–13.4)	9.5 (6.1–12.5)	3.3 (0–5.6)	12.3 (6.2–13.9)	8.5 (0–13.1)	5.1 (4.6–5.9)	0 (0–3.8)

Serial samples were separated into three groups: patients with Day 0, 5, and 28 samples; patients with Day 0 and Day 5 samples; and patients with Day 5 and Day 28 samples. N/A = not applicable.

**Table 3:** Multivariable linear regression model comparing viral loads between norovirus and rotavirus, and between norovirus and adenovirus.

	Baseline Viral Load		Day 5		Day 28	
	Adjusted Mean Difference of Log 10 Copies of Viral Load (95%CI)	P	Adjusted Mean Difference of Log 10 Copies of Viral Load (95%CI)	P	Adjusted Mean Difference of Log 10 Copies of Viral Load (95%CI)	P
Virus infected		Overall significance <0.0001		Overall significance <0.0001		Overall significance <0.0001
Rotavirus	1.77 (0.86, 2.68)	<0.0005	−0.93 (−1.65, −0.22)	<0.05	−2.59 (−4.06, −1.13)	<0.001
Adenovirus	1.84 (0.95, 2.73)	<0.0001	−1.53 (−2.25, −0.8)	<0.0001	−4.28 (−5.6, −2.95)	<0.0001
Norovirus	reference		reference		reference	
Age (per 1 month older)	−0.01 (−0.05, 0.02)	0.417	−0.006 (−0.03, 0.02)	0.640	−0.09 (−0.14, −0.03)	<0.005
Baseline duration of illness (per 1 hour greater)	−0.001 (−0.02, 0.02)	0.912	−0.024 (−0.04, −0.01)	<0.005	−0.001 (−0.03, 0.03)	0.955
Baseline MVS score (per 1 score greater)	0.23 (0.02, 0.43)	0.029	0.11 (−0.04, 0.27)	0.159	0.22 (−0.08, 0.52)	0.160
Baseline viral load (per 1 unit in log copies greater)	N/A	N/A	0.46 (0.34, 0.58)	<0.0001	0.41 (0.12, 0.7)	<0.01

MVS = Modified Vesikari Scale; N/A = not applicable.

**Table 4:** The viral load of stool samples in each patient with co-infection of mixed viruses.

Virus Detected	Viral Load (Log GE Copies/g Stool)								
	Adenovirus			Rotavirus			Norovirus		
	Day 0	Day 5	Day 28	Day 0	Day 5	Day 28	Day 0	Day 5	Day 28
Patient 1	13.2	11.5	-	6.6	0	-	-	-	-
Patient 2	13.3	10.4	-	10.3	13	-	-	-	-
Patient 3	5.6	6.1	0	12.9	12.5	4.4	-	-	-
Patient 4	6.2	5.0	0	12.3	11.1	0	-	-	-
Patient 5	-	-	-	13.1	11.4	-	5.5	0	-
Patient 6	-	-	-	12.7	6.4	-	6.5	0	-
Patient 7	-	-	-	12.9	7.9	4.1	6.6	6.3	0
Patient 8	-	-	-	0	11.8	5.6	10.3	0	0
Patient 9	4.3	5.2	-	-	-	-	10.8	9.1	-
Patient 10	5.6	0	-	-	-	-	9.5	10.2	-

“-” = either no sample collected or no virus detected.

## Data Availability

Not applicable.

## Supplementary Materials

The following supporting information can be downloaded at: https://www.mdpi.com/article/10.3390/v15071541/s1, Figure S1: Viral load of norovirus, rotavirus and adenovirus in each individual child who had Day 0, 5 and 28 serial samples: (A) Norovirus; (B) Rotavirus; (C) Adenovirus.
