# Supplementary material for: Significantly Longer Shedding of Norovirus Compared to Rotavirus and Adenovirus in Children with Acute Gastroenteritis
Source: Viruses. 2023 Jul 13;15(7):1541. doi: 10.3390/v15071541 (PMC10386448; doi:10.3390/v15071541)
Supplement: Supplementary file 1 [file viruses-15-01541-s001.zip › viruses-2467244-supplementary.pdf]

A.

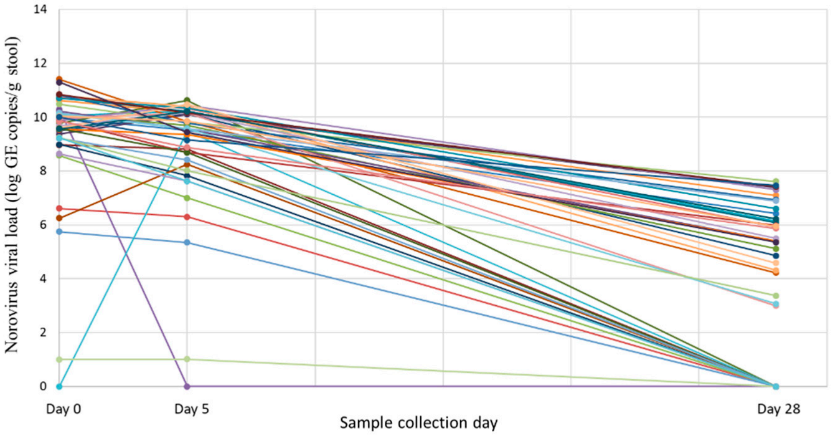

B.

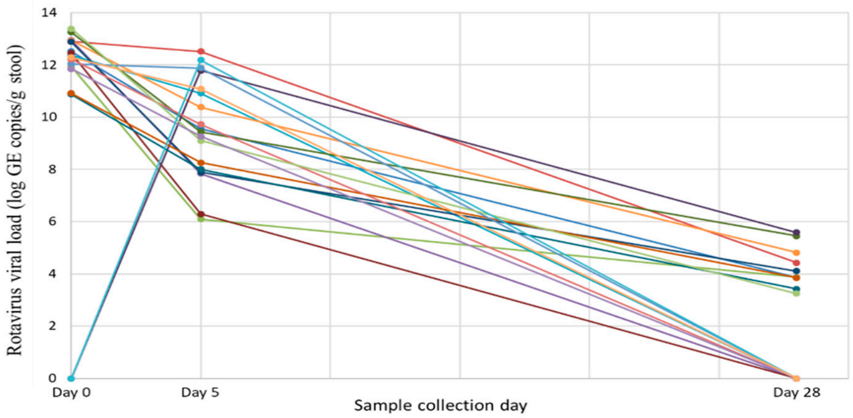

C.

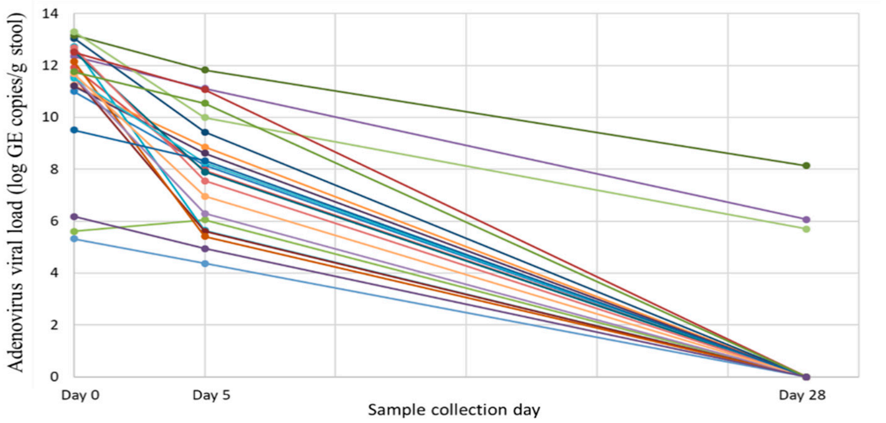

Supplemental Figure S1. Viral load of norovirus, rotavirus and adenovirus in each individual child who had Day 0, 5 and 28 serial samples: A) Norovirus; B) Rotavirus; C) Adenovirus. Each line refers to an individual child. The dot on Day 0, 5 and 28 represented the viral load of log GE copies/g of stool.
